# Simultaneous bioremediation of Disperse orange-2RL Azo dye and fatty acids production by *Scenedesmus obliquus* cultured under mixotrophic and heterotrophic conditions

**DOI:** 10.1038/s41598-022-22825-6

**Published:** 2022-12-01

**Authors:** Ragaa A. Hamouda, Noura El‑Ahmady El‑Naggar, Ghada W. Abou-El-Souod

**Affiliations:** 1 Department of Microbial Biotechnology, Genetic Engineering and Biotechnology Research Institute (GEBRI), University of Sadat City, Sadat City, Egypt; 2 Department of Biology, Faculty of Sciences and Arts Khulais, University of Jeddah, Jeddah, Saudi Arabia; 3 Department of Bioprocess Development, Genetic Engineering and Biotechnology Research Institute, City of Scientific Research and Technological Applications (SRTA-City), New Borg El‑Arab City, 21934 Alexandria Egypt; 4 Department of Botany and microbiology, Faculty of Science, Menoufia University, Shibin Al Kawm, Menoufia Egypt

**Keywords:** Biotechnology, Microbiology, Environmental sciences

## Abstract

Several types of green photosynthetic microalgae can grow through the process of heterotrophic growth in the dark with the help of a carbon source instead of the usual light energy. Heterotrophic growth overcomes important limitations in the production of valuable products from microalgae, such as the reliance on light, which complicates the process, raises costs, and lowers the yield of potentially useful products. The present study was conducted to explore the potential growth of green microalga *Scenedesmus obliquus* under mixotrophic and heterotrophic conditions utilizing Disperse orange 2RL Azo dye as a carbon source to produce a high lipid content and the maximum dye removal percentage. After 7 days of algal growth with dye under mixotrophic and heterotrophic conditions with varying pH levels (5, 7, 9, and 11), KNO_3_ concentrations (1, 1.5, 2, and 3 g/L), and dye concentrations (20, 40, and 60 ppm); dye removal percentage, algal dry weight, and lipid content were determined. The results showed that the highest decolorization of Disperse orange 2RL Azo dye (98.14%) was attained by *S. obliquus* in heterotrophic medium supplemented with glucose at the optimal pH 11 when the nitrogen concentration was 1 g/L and the dye concentration was 20 ppm. FT-IR spectroscopy of the dye revealed differences in peaks position and intensity before and after algal treatment. *S. obliquus* has a high concentration of oleic acid, which is enhanced when it is grown with Disperse orange 2RL Azo dye, making it ideal for production of high-quality biodiesel. In general, and in the vast majority of instances, heterotrophic cultivation is substantially less expensive, easier to set up, and requires less maintenance than mixotrophic cultivation. Heterotrophic cultivation allows for large-scale applications such as separate or mixed wastewater treatment along with biofuel production.

## Introduction

Synthetic dyes could lead to strong contamination of the environment and ecology due to the complex aromatic molecular contents of these dyes that are hard to degrade^1^. Many studies have shown that certain types of dyes, mainly azo dyes and their by-products, have adverse influences on humans and local biota^2^. Disperse orange 2RL azo dye induced cytotoxicity in Hep G2 cells^2^. Biological management of dyes with microorganisms is relatively cost-effective and eco-friendly due to minor sludge production^3,4^.

Many studies have reported the effectiveness of microalgae in the degradation of azo dyes^5–9^. The growth of microalgae in the textile dye effluent has been recognised as a favourable alternative to conventional methods of wastewater treatment. The treatment by microalgae diminishes the color and nutrient load of textile effluent, which diminishes numerous negative environmental influences^10^. Indigenous microalgae have undeniably the potential to rapidly, efficiently, and effectively eliminate dyes in wastewater to acceptable ranges of guide line limits at ambient temperature and neutral pH range^11^. Microalgae, which have been widely cultivated in wastewater treatment, have the ability to remove nutrients from a wide range of wastewater types, including industrial wastewater^12,13^.

Microalgae can live in a broad range of habitats and can be grown in three cultivation circumstances: autotrophic, heterotrophic, or mixotrophic. Autotrophic microalgae consume energy from light during photosynthesis processes to grow; mixotrophic microalgae can use organic carbon and light energy in growing; but in the case of heterotrophic; microalgae grow under dark conditions and use organic carbon as a carbon source^14^. Generally, microalgae are considered photoautotrophic microorganisms; meanwhile, also microalgae can grow in heterotrophic conditions, which can consume outdoor carbon sources with dark conditions, to acquire high-value products^15^. The biomass and lipid yield were highest with algae that grown in heterotrophic compared with phototrophic conditions, and also the cultivation of algae under the heterotrophic system is easier and cheaper to maintain large scale^16–18^.

Microalgae produce renewable energy because they can produce a high amount of oil in the body's cells. The oil composition in microalga ranges from 20 to 50% and microalgae dry weight yields can exceed 80%^19^. Biodiesel is organized through the trans-esterification of triglycerides or the esterification of free fatty acids (FFAs) with low-molecular-weight alcohols^20^. Biodiesel has minor exhaust emissions and toxicity compared to petroleum diesel fuel. It can be cast-off with conservative diesel as a mix or used on its own without modification^21^.

The present study was carried out to determine the potential removal of Disperse orange 2RL Azo dye by *S. obliquus* when grown under mixotrophic and heterotrophic conditions with and without an amended carbon source to the medium, and to determine the best pH, KNO_3_ and dye concentrations to obtain high removal percentage of dye and maximum production of lipids content to produce fatty acid methyl ester (FAME) biodiesel.

## Material and methods

### Dye and chemicals

**Figure 1:**
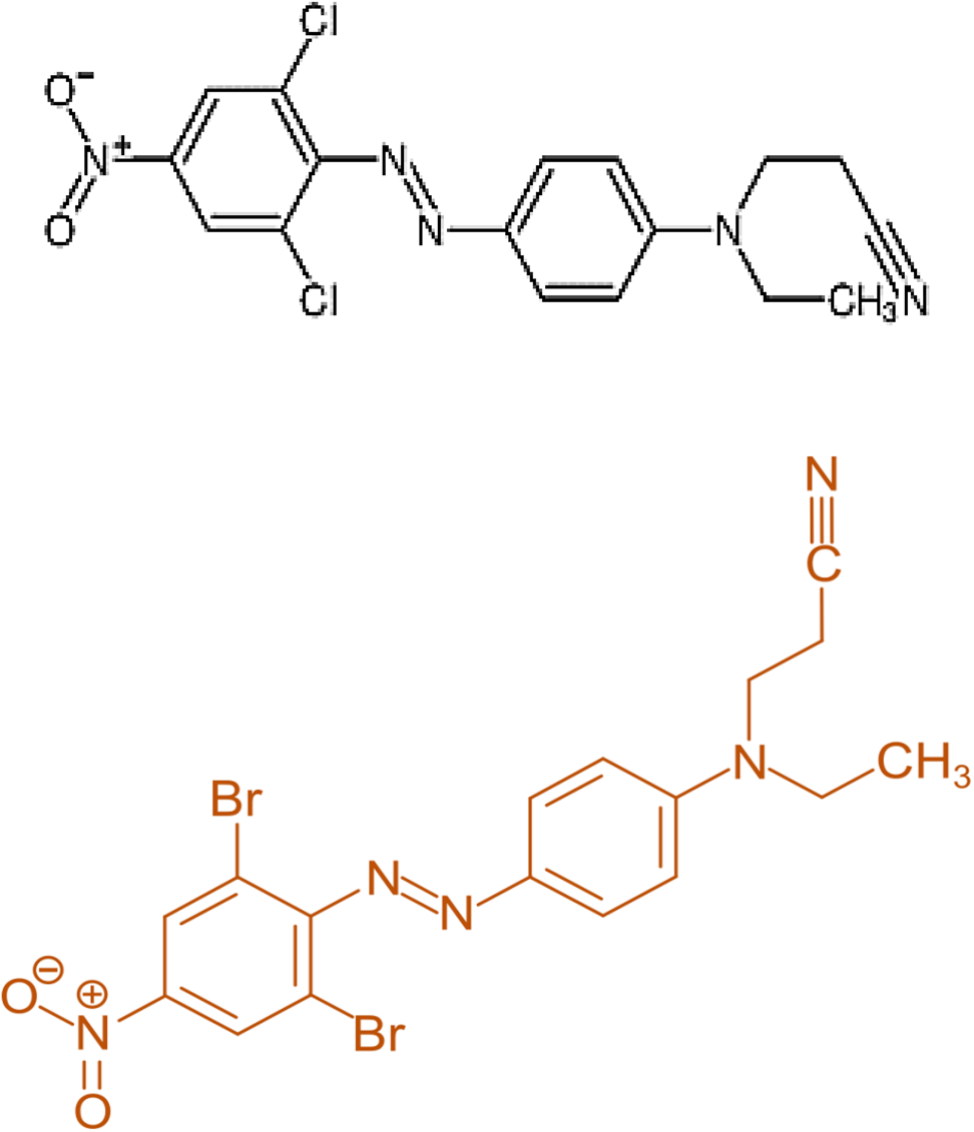
Disperse orange 2RL.

*Scenedesmus obliquus* microgreen alga was acquired from the ‟Microbial Biotechnology Department, Genetic Engineering and Biotechnology Research Institute (GEBRI), University of Sadat City, Egypt”. Disperse orange 2RL (Disp.orange 2RL (4-nitro-4′-[N-ethyl-N-(2-cyanoethyl)-amino [azo benzene), it was obtained from Dyeing Factory at the industrial region district in Quisna, Menoufia Governorate, Egypt as Fig. 1.

### Alga culture

*Scenedesmus obliquus* was grown with small modified bold Basal medium^22^.

### De-colorization study and spectroscopic analysis

Different concentrations 0, 20, 40, and 60 ppm of Disperse orange 2RL (4-nitro-4′-[*N*-ethyl-N-(2-cyanoethyl)-amino [azo benzene) were adjusted in 150 mL of bold Basal medium and alga (120 mL and 30 mL alga culture in stationary phase), the culture was incubated at 25 °C for 7 days. For mixotrophic conditions the culture was exposed to continuous light with intensity of 80 μE m^−2^ s^−1^ continuous light, while for heterotrophic conditions, the cultures were kept in dark.

### Effect of glucose

The effect of glucose on the dye removal by alga was tested by the addition of 5% glucose to the medium (Heterotrophic with glucose).

### Effect of pH

pH levels were adjusted at 5, 7, 9, and 11, and applied in various conditions mixotrophic, heterotrophic and heterotrophic with media supplemented with 5% glucose. The concentration of dye was adjusted at 20 ppm.

### Effect of nitrogen concentrations

Bold Basal medium supplemented with different concentrations of KNO_3_ (1, 1.5, 2, and 3 g/L) to test the decolorization % of dye with concentrations 20 ppm, by alga under mixotrophic and heterotrophic and heterotrophic supplemented with 5% glucose.

### Decolorization percentage

After 3, 5, and 7 days of incubation, the 15 ml of algal suspensions were centrifuged, and measuring the absorbance of the cell-free supernatant of the sample by Spectrophotometric UV–Vis Dual Beam UVS-2700 at 429 nm. The percentage of decolorization was calculated by using the equation as follows according to Telke et al.^23^.

$${\text{Decolorization}}\;\left( \% \right) = \frac{{{\text{Initial}}\;{\text{absorbance}}{-}{\text{Final}}\;{\text{absorbance}}}}{{{\text{Initial}}\;{\text{absorbance}}}} \times 100$$

### Dry weight estimation

Following the completion of the incubation period of 3, 5, and 7 days, aliquots of 150 ml were centrifuged, washed three times with distilled water, and the residue was dried till constant weight in an oven at 60 °C until constant weight was achieved.

### Lipids determination

One gram of algal dry weight was mixed with 50 mL of methanol and then stirred with a magnetic stirrer for 20 min. The residue was then separated by filtration, and the solvent had been evaporated. The extracted lipid was determined in percentage^7^, using the following equation:

$${\text{Lipid}}\;\% = \left( {{\text{G}}_{1} {\text{/G}}_{2} } \right)*100$$

where G_1,_ lipids weight, G_2_ dry weight.

### Infrared measurements (IR)

After 7 days of incubation, the biodegraded dye samples were characterized before and after treatment using FTIR spectroscopy (Bruker). The analyses of the biodegraded dyes were compared with the control dyes. The FTIR analysis was done in the mid IR region (500–3500 cm^−1^)^5,24,25^. The infrared measurements demonstrated in decolonization at 20 ppm Disperse orange 2RL by *S. obliquus* was (98.14%), when nitrogen concentration was 1 g/l. The highest decolorization of Disperse orange 2RL was attained by *S. obliquus* in heterotrophic with glucose at pH 11.

### Preparation of fatty acid methyl ester (FAME) content and analysis by GC–MS

20 mg of the lipid extract were weighted and then 0.5 N methanolic KOH (2.805 gm KOH in 100 mL methanol) were added, vortex and heat at 50 °C for 15 min. The mixture was then cooled and vortex mixed. Then 5 ml of 0.4 N HCL (3.34 ml HCL in 100 mL water) were added and vortex mixed. 5 ml of petroleum ether and hexane, 1:1 was then added. The upper layer (FAME) was transferred to another tube and dried at 40 °C. One mL of hexane was then added to the dried FAME. The FAME was then injected and analyzed by GC–MS. The sample was injected in GC–MS with silica capillary column HP-5MS, the carrier gas was Helium. The GC–MS temperature program was initiated at 60 °C (2 min) and then increased to 280 °C at an ionizing rate of 8 °C/min. To evaluate the different peaks, Wiley and Wiley Nist mass spectral databases were applied^26–28^.

### Statistical analysis

Statistical analysis was performed using ANOVA, by using SPSS software (version 19) for comparison between treatments according to Sokal and Rohlf^29^. Significant differences between means of parameters were determined by using Duncan’s multiple range tests with probability ≤ 0.05.

## Results and discussion

### Effects of different concentrations of Disperse orange 2RL on the efficiency of removal percentage of dye by *S. obliquus* with time under mixotrophic conditions

**Figure 2:**
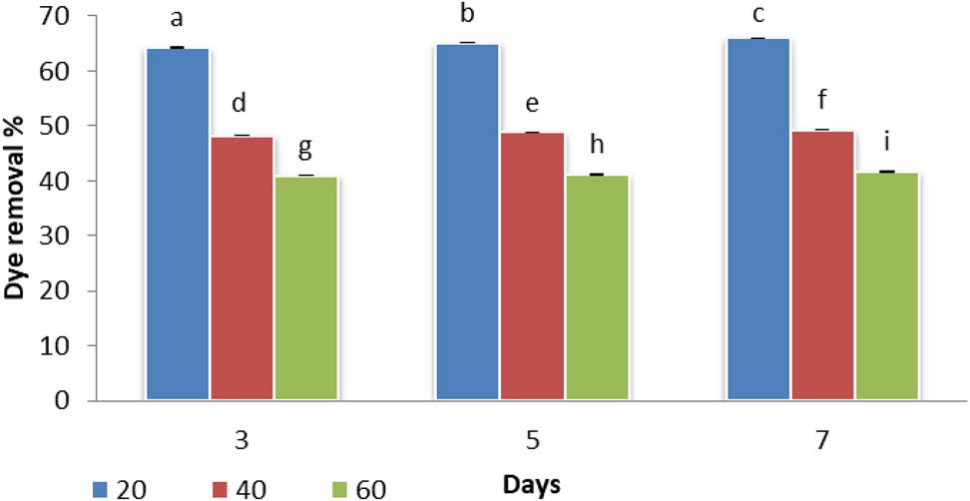
Effect of different concentrations of Disperse orange 2RL on removal percentage by microgreen alga *S. obliquus* with days under mixotrophic conditions, Par represent standard error, different letters clear the significant result.

The degree of decolorization of Disperse orange 2RL by *S. obliquus* was studied at different concentrations of dye (20, 40, and 60 ppm) with 3, 5, and 7 days, the batch cultures were incubated in continuous lights. Figure 2 shows that there was an increase in the decolorization rate with increasing the incubation time. The results show *S. obliquus* has the maximum percentage of degradation after treatment with Disperse orange 2RL (20 ppm) of 64.31%, 65.15% and 66.01% after 3, 5 and 7 days of incubation, respectively. The less degradation of Disperse orange 2RL by *S. obliquu*s occurred at 60 ppm. These findings are consistent with those of Chen et al.^30^, who found that dye decolorization by microorganisms could be owing to adsorptive by algae biomass or biodegradation by algae. With different initial dye concentrations, the quantity of color loss varies. Algae and microorganisms removed color in a concentration-dependent manner, which was attributed to bioconversion^30,31^. Bafana et al.^32^ concluded in their study that the decolorization of azo dyes can be related to biodegradation.

### Effect of different conditions on dye removal by *S. obliquus*

#### pH levels

**Figure 3:**
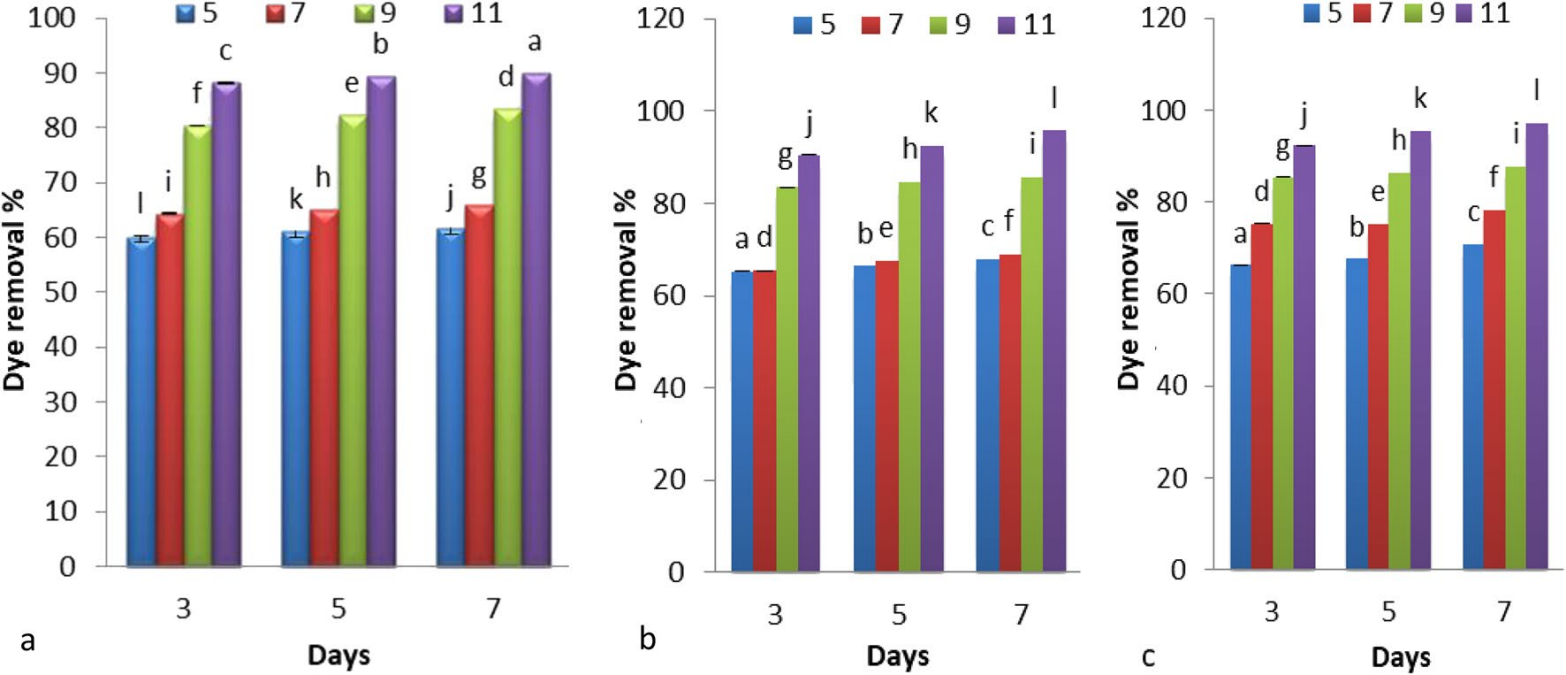
Effect of different pH levels on the Disperse orange 2RL (20 ppm) removal percentage by microgreen alga *S. obliquus* grown under Mixotroph (**a**), Heterotroph (**b**), Heterotroph supplemented with glucose (**c**). Par represent standard error, different letters clear the significant result.

Figure 3 represents the effect of different pH levels on the Disperse orange 2RL removal percentage by microgreen alga *S. obliquus* grown under mixotrophic, heterotrophic, and heterotrophic culture supplemented with glucose. The maximum decolorization of Disperse orange 2RL by *S. obliquus* with the mixotrophic condition was 89.94% at 7 days of growth, in case of heterotrophic culture was 95.73%, and in heterotrophic culture with glucose was 97.23%. The decolorization of Disperse orange 2RL by *S. obliquus* in heterotrophic culture with glucose at pH 11 was achieved the maximum removal. The removal rate of Reactive Red 3B-A and Reactive Black 5 by *Clostridium bifermentans* strains was increased when the addition of glucose^33^. Carbon sources provide the necessary energy for the proliferation of microorganisms and also function as electron donors, which are necessary for the rupturing of the azo bond^34^. The best level of pH for all growth conditions was 11. The ability of algae to remove azo dye depends on the pH levels on the medium and the stability of pH in the oxidative enzymes. El-Sheekh et al.^6^ reported that the best pH to remove azo dye by *S. obliquus* was 11, due to alga exerted some acidic compounds that released to medium, which neutralize the high pH levels of the medium. Chen et al.^35^ concluded that the optimum pH levels for high removal efficiency of azo dyes are often ranged from 6 to 10 for most dyes. Under the alkaline condition, azo dyes lose hydrogen ions, which cause ionization of the dye, influence the stability, and help the removal of dye from solutions^36^. The optimum pH level for removal Direct Blue 71 and Disperse Red 1 by *Chlorella vulgaris* was found to be at 8^1^. Increasing in pH from 6 to 8 caused important proliferation in the rate of removal dye with indigenous microalgae^11^.

#### Nitrogen concentrations

**Figure 4:**
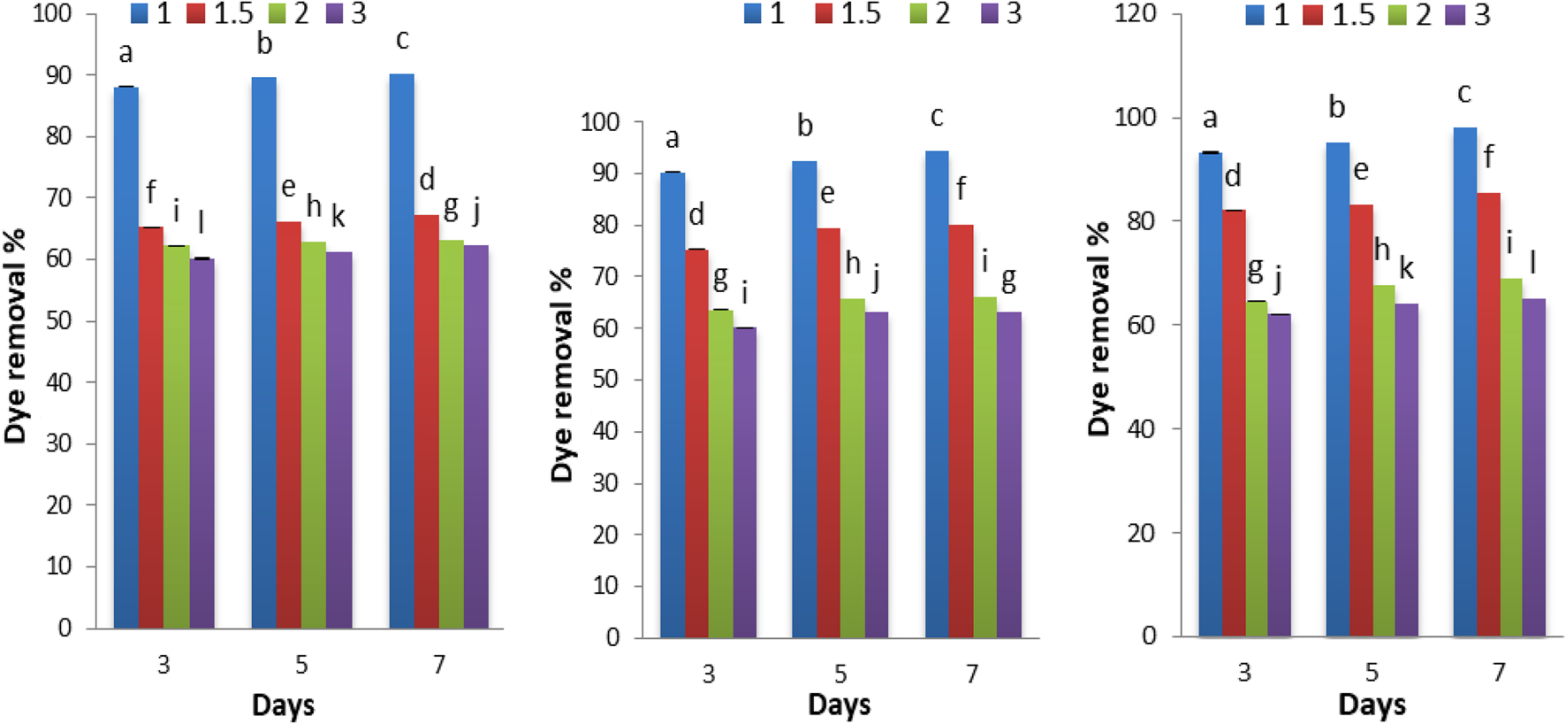
Effect of different nitrogen concentrations on the of Disperse orange 2RL (20 ppm) removal percentage by microgreen alga *S. obliquus* grown under mixotrophic (**a**), heterotrophic (**b**), heterotrophic supplemented with glucose (**c**). Par represent standard error, different letters clear the significant result.

Figure 4 displays the degradation of Disperse orange 2RL by *S. obliquus* at different nitrogen concentrations (1, 1.5, 2, and 3 g NaNO_3_/L) supplemented to the algal medium. The maximum percentage of dye removal percentage (98.14%), when nitrogen concentration was 1 g/L and was obtained when alga is grown under heterotrophic with glucose at 7 days, meanwhile 90.30% with mixotrophic, 94.35% with heterotrophic without glucose. The addition of nitrogen and phosphorus to the liquid media triggered biodegradation^37–39^.

### Effects of different concentrations of Disperse orange 2RL on *S. obliquus* dry weight

**Figure 5:**
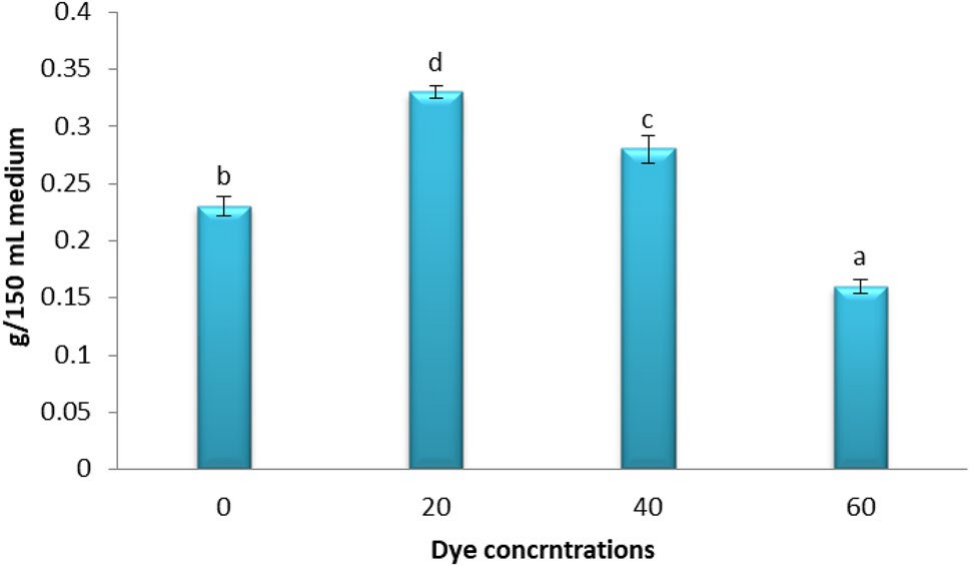
Effect of different dye concentrations of the of Disperse orange 2RL on alga (*S. obliquus*) dry weight with mixotrophic conditions, pH 7 and KNO_3_ 1.5 g/L. Par represent standard error, different letters clear the significant result.

The maximum decolorization of different dye concentrations of the of Disperse orange 2RL on alga dry weight at 20 ppm was 0.33 g/L with pH 7, KNO_3_ 1.5 g/L, under mixotrophic conditions. The increasing of dye concentrations led to a decrease in the dry weight of alga Fig. 5. The present results agree with the previous findings of Acuner and Dilek^40^ concluded that high concentrations of dye had negative effects on algal growth. The growth rate of the *Scenedesmus quadricauda* decreased as indigo dye effluent concentration increased^41^. Algae undoubtedly have the potential to rapidly, efficiently, and effectively remove dyes to low concentrations and less toxic compounds^42^. According to the findings, the chosen microalga is capable of degrading and removing the color of various dyes from wastewater effluents. These findings are consistent with those of Anjaneyulu et al.^43^, who found that microalgae may remove dye color by various processes of assimilative chromophore usage for the generation of algal biomass, CO_2_, and H_2_O. As a result, colored dye molecules convert into non-colored ones, and chromophore adsorption on algal biomass occurs.

### Effect of different conditions on the algal dry weight grown with Disperse orange 2RL

#### pH levels

**Figure 6:**
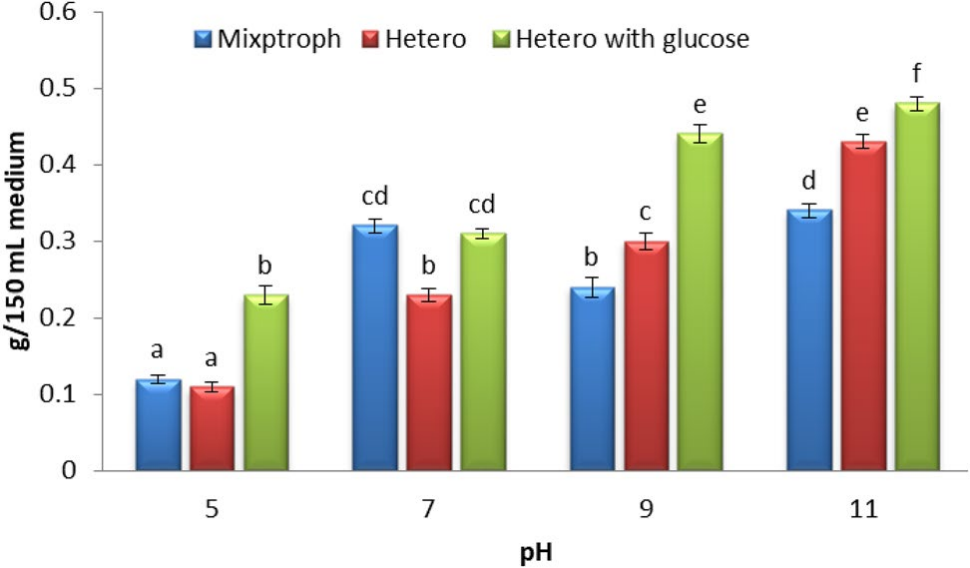
Effect of different pH levels on the dry weight of alga (*S. obliquus*) grown with the Disperse orange dye (20 ppm) under different growth conditions. Par represent standard error, different letters clear the significant result.

The maximum algal dry weight **(**Fig. 6) in the Disperse orange at pH 11 was obtained with mixotrophic (0.34 g), heterotrophic without glucose (0.43 g), heterotrophic with glucose was (0.48 g). In addition to having an effect on the cellular growth of algae, the pH had an effect on the enzyme activity that was responsible for the removal of the dye^44^. At high pH values, there was an increase in the amount of protein and biomass produced by *S. obliquus*^45^. The highest biomass yields were obtained when *Scenedesmus* sp. was cultivated at pH 8^46^. The maximum biomass and chlorophyll content were obtained when *Chlorella vulgaris* was grown at pH 11^47^.

#### KNO_3_

**Figure 7:**
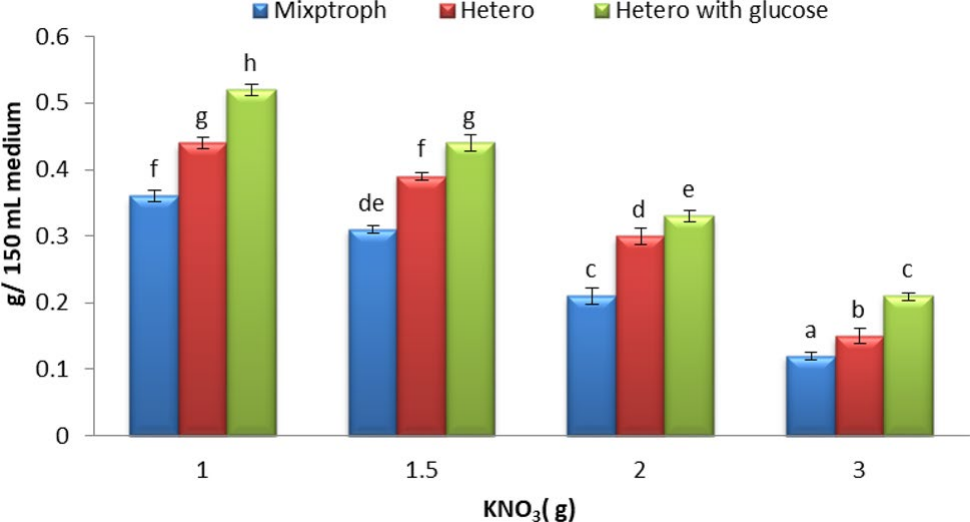
Effect of nitrogen concentrations in dry weight of alga (*S. obliquus*) was grown with the of Disperse orange dye (20 ppm) under different growth conditions. Par represent standard error, different letters clear the significant result.

The results in Fig. 7 represent the maximum *S. obliquus* dry weight (0.52 g) obtained with 1 g KNO_3_/liter of the medium, when was grown in heterotrophic conditions and medium supplemented with glucose, followed by 0.44 g with heterotrophic, 0.36 g with mixotrophic. Glucose is the greatest commonly consumed carbon source for heterotrophic cultures of microalgae, as is the case for many other microbial species. Maximum rates of growth and respiration are attained with glucose than with any other sugars^48^. Glucose-induced physiological alterations in *Chlorella vulgaris*, which have a significant impact on carbon assimilation metabolic pathways, cell size, and storage material volume densities, such as starch and lipid grains^49^. Zili et al.^50^ reported that amended of glucose to the algal medium of *Graesiella* sp. promoted biomass and lipid productivity. Glucose was the best sugar among five sugar were tested that enhancement *Scenedesmus* sp. LX1density and biomass^51^.

### Effect of dye concentrations on lipid percentage of alga

**Figure 8:**
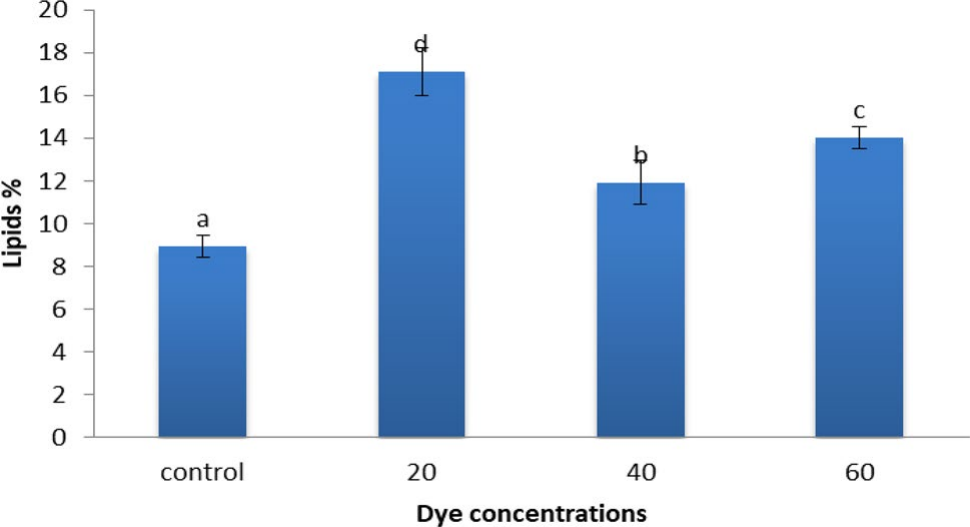
Effect of different dye concentrations (Disperse orange 2RL) on lipids percentage produced by microgreen alga *S. obliquus,* under mixotrophic conditions, pH 7 and KNO_3_ 1.5 g/L. Par represent standard error, different letters clear the significant result.

**Figure 9:**
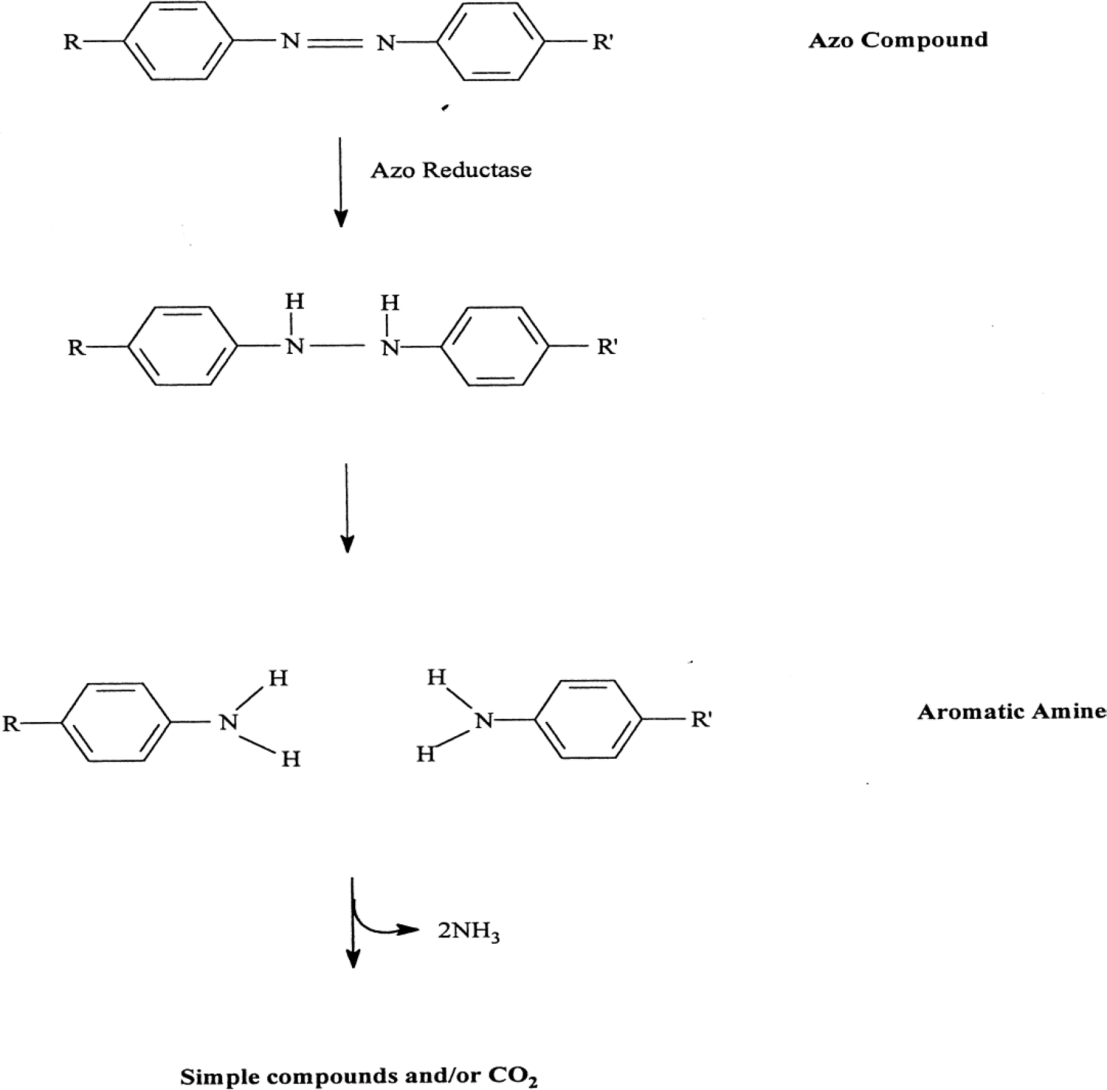
Degradation of azo dyes by algae.

The data present in Fig. 8 denotes the lipid percentage content was 17.09, 11.92, and 14.03 of *S. obliquus* was grown with 20, 60 and 40 ppm Disperse orange 2RL respectively with mixotrophic conditions. Dye concentration 20 ppm was the best concentrations for enhancement lipid productions of *S. obliquus.* So this concentrations was chosen to complete the experiments, and test the effects of different conditions such as mixotrophic, heterotrophic and heterotrophic supplemented with 5% glucose with various pH levels, and various KNO_3_ on lipid algal contents to possible productions high amount of lipids, and biodiesel yields. Three different mechanisms of decolorization dyes by algae, adsorption of chromophore on algal biomass, degrading azo dyes, through an induced form of an azo reductase, degrading azo dyes, through an induced form of an azo reductase, utilization of chromophore for production of algal biomass, CO_2_ and H_2_O transformation of colored molecules to non-coloured molecules^43^. Figure 9 represents the degradation of dye. The carbon dioxide produced through decolorization of dye is utilized in lipid production by algae. Enrichment of CO_2_ as inorganic carbon source stimulated lipid biosynthesis and cell growth by accelerating photosynthetic carbon fixation in microalgae^52^. Neutral lipid production and accumulation was strongly accelerated in the presence of exogenous organic carbon source by accompanying with abolishing chlorophylls in a unicellular green alga *Chlorella protothecoides*^53^.

### Effect of different conditions pH and KNO_3_ on lipids percentage produced by *S. obliquus* was grown with Disperse orange 2RL under mixotrophic and heterotrophic with and without glucose

**Figure 10:**
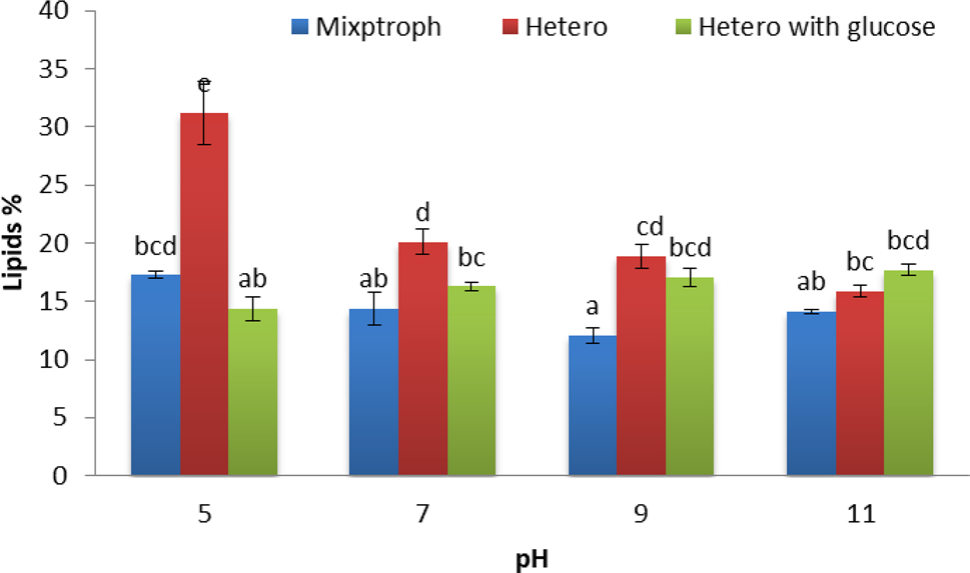
Effect of different pH levels on lipids percentage produced by microgreen alga *S. obliquus* that grows with Disperse orange 2RL (20 ppm) at different conditions. Par represent standard error, different letters clear the significant result.

**Figure 11:**
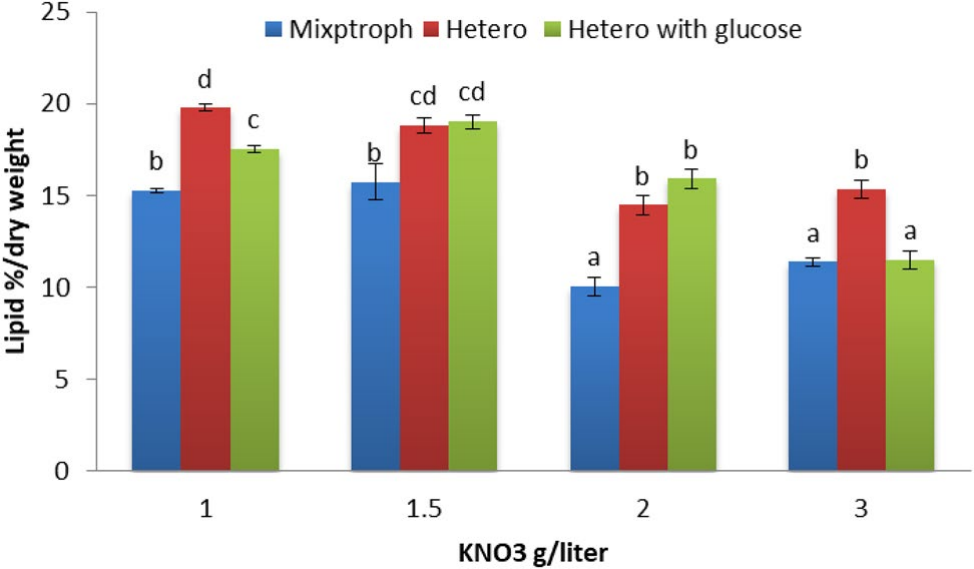
Effect of different nitrogen concentrations Disperse orange 2RL (20 ppm) on lipids percentage produced by microgreen alga *S. obliquus* that was grown at different conditions. Par represent standard error, different letters clear the significant result.

The yield of microalga (*Scenedesmus* sp.) lipid per unit area was increased when grown under heterotrophic conditions with wastewater^54^. Figure 10 displays the effect of different pH levels on lipids percentage produced by microgreen alga *S. obliquus* that was grown with Disperse orange 2RL at different conditions. Results denote the best pH level was 5 which gives 31.16% of lipid produced by *S. obliquus* in heterotrophic condition, and also heterotrophic condition was denoted the best results at pH 7 and 9 in comparison with mixotrophic and heterotrophic with glucose. The results demonstrate there were significant effects at each level of pH among different conditions. The efficacy of azo dye removal is mostly determined by the pH in the middle, the endurance of azo dye analyzers, and the pH stability of oxidative enzymes. Any components of the microalgae cell carry out their critical duties under a specified pH, according to microalgae physiology and dye ingredients. The production of acidic extracellular metabolites by microalgae contributes to the neutralization of the high alkaline pH. This is one of the ways in which microalgal cells will defend themselves. Any portions of the cell microalgae (thylakoid of chloroplasts) conduct critical tasks under a particular degree of acidity due to the physiology of micro-members of microalgae. The photosynthetic process is significantly affected by the pH of the medium in which the microalgae are growing, which can have a considerable influence on the process. The alkaline medium is used for producing algae since the ideal pH value for microalgae growth is between 8.2 and 8.7^6^, increasing the yield of microalgal lipid per unit area. Figure 11 exhibits the effect of different nitrogen concentrations (Disperse orange 2RL) on lipids percentage produced by microgreen alga *S. obliquus* that was grown at different conditions. The best result was obtained at heterotrophic conditions (19.77%) with 1 g KNO_3_, followed by 1.5 g KNO_3_ at heterotrophic culture supplemented with glucose. No significant effects were produced in lipid percentage at 1.5 and 2 g KNO_3_ between heterotrophic and heterotrophic with glucose. The starvation of nitrate leads to the accumulation of lipids, and also changes in fatty acids contents^55^. The starvation of KNO_3_ leads to the accumulation of lipids in microgreen alga *S. obliquus*^56^.

There are three limiting factors in the degradation process of azo dyes by algae: (1) Dye degradability, which is mostly determined by the structural characteristics of dyes. (2) Effects of ambient circumstances on algae's ability to use dyes, which was assessed by algal physiological features^57^. In the current study, it was found that alga has the ability to break down azo dye, and that this ability is controlled by the physiological characteristics of the alga and the structure of the dye. These findings are consistent with those of Chen et al.^35^, who found that dye decolorization by microorganisms could be owing to adsorption to algae biomass or biodegradation by algae. The amount of dye decolorization can vary depending on the initial concentration of the dye^58^.

### Infrared measurements (IR)

**Figure 12:**
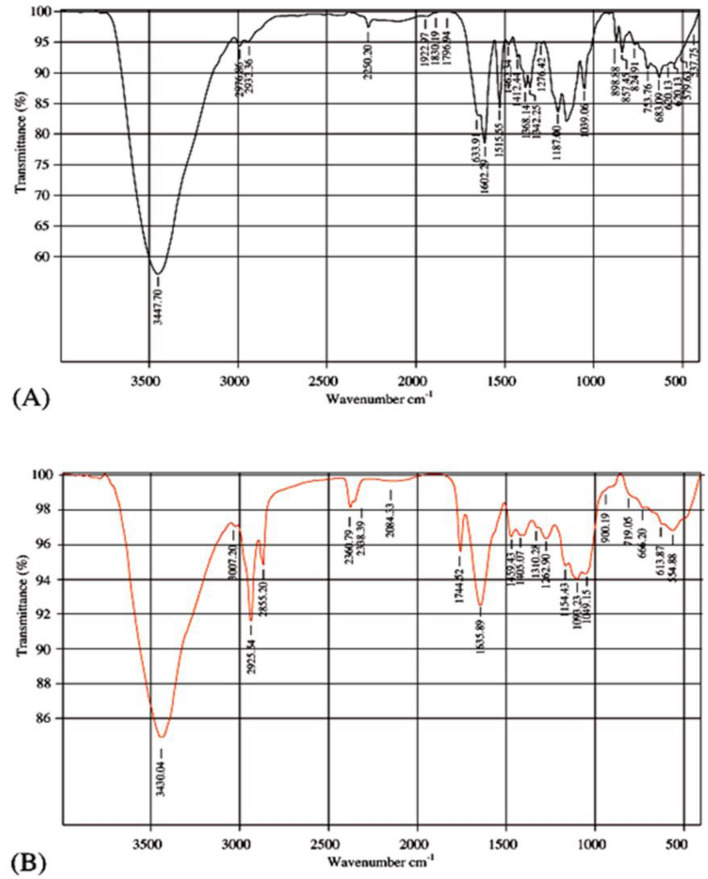
Infrared spectrum of Disperse orange before *Scenedesmus obliquus* action (**A**) Infrared spectrum of Disperse orange after *Scenedesmus obliquus* action (**B**).

The results in Table 1 and Fig. 12 denote the infrared analysis of Disperse orange 2RL before and after treatments with *S. obliquus*. The results were evident; there were changes in azo region. The measured changes in spectral intensity and the existence of stretched vibration in IR of algal biomass treated with dyes revealed probable bio-sorption in addition to algal degradation activities in the current study. To show the intensity of the IR peak and the change in the structure of the dye compound before and after treatment by the alga. The peaks 1620.29 cm^−1^ and 1515 cm^−1^ presented in Disp. orang 2RL, but disappeared when treated with alga. Peak 1633.91 cm^−1^ in Disp. orang 2RL shifted to 1635 cm^−1^ after treatment by *S. obliquus*. There were emerged new peaks after treatment with *S. obliquus* 1635.89 cm^−1^ and 1744.52 cm^−1^ in azo range. The peak at 1515 cm^−1^ in Disperse orange disappeared after treatment with *S. obliquus* So, there is changes in azo range before treatments compared to after treatments with *S. obliquus*., the changes were achieved in Disperse orange after treatment with *S. obliquus* in azo range, so these results suggest that algal action cause the cleavage of azo linkage of Disp.orange2RL. These results confirmed the results of Disp.orange 2RL degradation, where high percentage was reached at 98.14%, by *S. obliquus* when nitrogen concentration was 1 g/l. The highest decolorization of Disperse orange 2RL was attained by *S. obliquus* in heterotrophic with glucose at pH 11 after 7 days of incubation. These results may be due to the ability of this alga to induce azo reductase enzyme under azo dye stress condition, these results agree with that obtained by Urushigawa and Yonezawa^89^.

**Table 1:** Infrared spectrum of Disperse orange before *S. obliquus* action and Infrared spectrum of Disperse orange after *S. obliquus* action.

Disperse orange		Disperse orange after *S. obliquus* action		Difference	References
Wave Cm^−1^	Functional groups	Wave Cm^−1^	Functional group		
3447	Is attributed to –NH_2_ and –OH groups stretching vibration	3430	N–H stretching bands of mainly trans-ordered substructures	− 17	^59,60^
2976	C–H stretching band	3007	C–H	+ 31	^61^
2931	Stretching C–H	2925	C–H stretching	− 6	^62,63^
		2855	CH_2_ of lipids		^64^
		2360	C=O		^65^
2250	C≡N	2358	Corresponding to N–H	− 8	^66^
		2084	Stretching bands of C≡N		^67^
1922	(C=C=C)				^68^
1830	Stretching vibrations of C–O and C=O				^69^
1796	Strong C=O peak	1744	C=O stretching of phospholipids	− 52	^70,71^
1633	Amide I′ band	1635	Absorbed O–H and conjugated C–O in polysaccharides	+ 2	^72,73^
1620	Aromatic C=C stretching				^74^
1515	Secondary amine group				^75^
1460	Asymmetric CH_2_ bending	1459	Terminal methyl groups	− 1	^76,77^
1412	Stretching C–N,	1405	CH_3_ asymmetric deformation	− 7	^78,79^
1368	CH_2_ polysaccharides				^80^
1342	CH_2_ wagging vibrations	1310	Amide III	− 32	^81,82^
1276	N–H thymine	1262	PO_2_ asymmetric (Phosphate I)	− 14	^78^
1187	C–N stretching	1154	C–NH–C	− 33	^83^
		1093	C–O vibrations		^84^
1039	Si1O stretching of montmorillonite	1049	C–O of alkoxy	+ 10	^85,86^
898	C–O groups	900	Phosphodiester region	− 2	^87,88^

### Fatty acids methyl ester

The GC–MS analysis of Fatty acids methyl ester extracted from *S. obliquus* (control) after methylation was 10 compounds. These compounds are n-Hexadecanoic acid (C_16_H_32_O_2_ ), Oleic Acid (C_18_H_34_O_2_), Oleoyl chloride (C_18_H_33_ClO), 9-Octadecenoic acid (Z)-, 2,3-dihydroxypropyl ester (C_21_H_40_O), Yohimbic acid (C_20_H_24_N_2_O_3_), 9-Octadecenoic acid (Z)-, 2-hydroxy-1,3-propanediyl ester (C_39_H_72_O_5_), 1-Oxa-3-azaspiro[4.5]decan-2-one, 3-cyclohexyl-4-hydroxy-4-methyl (C_15_H_25_NO_3_), 0,11-Epoxy-n-undecan-1-ol (C_11_H_24_O), 9-Octadecenoic acid, 1,2,3-propanetriyl ester, (E,E,E)-(C_57_H_104_O_6_), and 8,12-Trimethyltridecan-4-olide (C_16_H_30_O_2_), the percentage area was 9.19, 40.77, 3.12, 1.69, 4.93, 1.31, 19.04, 1.74, 1.27 and 16.89 respectively Table 1. The lipids extracted from *S. obliquus* which was grown under heterotrophic conditions, with pH 11, medium supplemented with 1 g KNO_3_ and supplemented with 20 ppm Disperse orange 2RL Azo dye, were seven compounds, n-Hexadecanoic acid, Oleic Acid, Oleoyl chloride, 9-Octadecenoic acid (Z)-, 2,3-dihydroxypropyl ester, 9-Octadecenoic acid (Z)-, 2-hydroxy-1,3-propanediyl ester, Oleic acid, 3-(octadecyloxy) propyl ester and Urs-12-en-28-ol, the percentage of area was 18.44, 72.049, 1.72, 4.62, 1.3, 1.07 and 0.76 respectively Table 1. Unpaprom et al.^89^ reported *Scenedesmus acuminatus* had the highest oleic acid content, in comparison with the other five algae, which makes it the most suitable isolate for the manufacture of good quality biodiesel. This study indicated the heterotrophic conditions with 1 g KNO_3_ amended to algal medium and pH 11were the best conditions for lipids productions, and contents of *S. obliquus*. Li et al.^90^ reported that the highest lipid contents of *Chlorella protothecoides* were obtained when growing under heterotrophic cultivation in comparison to heterotrophy–photoinduction cultivation. Shen et al.^91^ reported that mixotrophic conditions with nitrogen depletion led to high FAME produced by *S. obliquus*. El‑Naggar et al.^92^ reported that *S. obliquus* proved efficiency in eliminating dyes and produce renewable fuels, such as biodiesel (Table 2).

**Table 2:** GC–MS analysis of Fatty acids methyl ester (FAME) produced by *S.obliquus* (a) and under grown with Disperse orange 2RL Azo dye under hetrotrophic conditions with medium supplemented with 5% glucose at pH 11 and KNO_3_ conc., 1 g/L (b).

Compounds	Area% of control	Area% after treatment
n-Hexadecanoic acid (C_16_H_32_O_2_)	9.19	18.44
Oleic Acid (C_18_H_34_O_2_)	40.77	72.049
Oleoyl chloride (C_18_H_33_ClO)	3.12	1.72
9-Octadecenoic acid (Z)-, 2,3dihydroxypropyl ester (C_21_H_40_O)	1.69	4.62
Yohimbic acid (C_20_H_24_N_2_O_3_)	4.93	–
9-Octadecenoic acid (Z)-, 2-hydroxy-1,3-propanediyl ester (C_39_H_72_O_5_)	1.31	1.3
1-Oxa-3-azaspiro[4.5]decan-2-one, 3-cyclohexyl-4-hydroxy-4-methyl (C_15_H_25_NO_3_)	19.04	–
0,11-Epoxy-n-undecan-1-ol C_11_H_24_O	1.74	–
9-Octadecenoic acid, 1,2,3-propanetriyl ester, (E,E,E)-C_57_H_104_O_6_	1.27	–
8,12-Trimethyltridecan-4-olide (C_16_H_30_O_2_)	16.89	–
Oleic acid, 3-(octadecyloxy) propyl ester (C_39_H_76_O_3_)	–	1.07
Urs-12-en-28-ol (C_30_H_50_O)	–	0.76
Total	98.22	99.95

## Conclusions

The experimental design strategy combined with the use of the desirability function for the optimization of nitrogen, pH, different concentration of dyes showed to be a successful tool for maximizing cell and oil production simultaneously. The high biomass and lipid production under nutrient deprivation and ambient air supply could decrease the cost of biodiesel production. Hence, the high proportion of C16 and C18 in the lipids showed *Scenedesmus* sp. to be a potential microalgae species for biodiesel production. When compared to phototrophic circumstances, algae that grow in a heterotrophic system produce the most biomass and lipid production, and the growth of algae in a heterotrophic system is both easier and less expensive to maintain on a large scale.

## Data Availability

The datasets spent and/or analyzed during this study are available from the corresponding author on reasonable request.
